# *Salmonella enterica* Serovar Typhimurium Travels to Mesenteric Lymph Nodes Both with Host Cells and Autonomously

**DOI:** 10.4049/jimmunol.1701254

**Published:** 2018-11-28

**Authors:** Alberto Bravo-Blas, Lotta Utriainen, Slater L. Clay, Verena Kästele, Vuk Cerovic, Adam F. Cunningham, Ian R. Henderson, Daniel M. Wall, Simon W. F. Milling

**Affiliations:** * Institute of Infection, Immunity and Inflammation, College of Medical, Veterinary and Life Sciences, University of Glasgow, Glasgow G12 8TA, United Kingdom;; † Institute of Immunology and Immunotherapy, University of Birmingham, Birmingham B15 2TT, United Kingdom; and; ‡ Institute of Microbiology and Infection, College of Medical and Dental Sciences, University of Birmingham, Birmingham B15 2TT, United Kingdom

## Abstract

*Salmonella* infection is a globally important cause of gastroenteritis and systemic disease and is a useful tool to study immune responses in the intestine. Although mechanisms leading to immune responses against *Salmonella* have been extensively studied, questions remain about how bacteria travel from the intestinal mucosa to the mesenteric lymph nodes (MLN), a key site for Ag presentation. In this study, we used a mouse model of infection with *Salmonella enterica* serovar Typhimurium (STM) to identify changes in intestinal immune cells induced during early infection. We then used fluorescently labeled STM to identify interactions with immune cells from the site of infection through migration in lymph to the MLN. We show that viable STM can be carried in the lymph by any subset of migrating dendritic cells but not by macrophages. Moreover, approximately half of the STM in lymph are not associated with cells at all and travel autonomously. Within the MLN, STM associates with dendritic cells and B cells but predominantly with MLN-resident macrophages. In conclusion, we describe the routes used by STM to spread systemically in the period immediately postinfection. This deeper understanding of the infection process could open new avenues for controlling it.

## Introduction

*Salmonella enterica* species present a serious public health burden, causing gastroenteritis and systemic infection in humans and domestic animals. *S. enterica* serovars Typhi and Paratyphi cause systemic fever in humans, with over 20 million cases and 200,000 deaths annually (1), including virulent *S. enterica* serovar Typhimurium (STM) strains that have emerged in sub-Saharan Africa with 20–25% mortality (2). STM infects a wide range of animals and is one of the most common serotypes associated with human infections. Experimentally, many genetic variants of this strain are available, including constructs expressing a wide range of reporter genes. These STM strains are used to induce acute or persistent infections with localized or systemic disease (3, 4) and have been used to identify molecular determinants controlling the infective process.

Following oral ingestion, STM can cross the intestinal epithelium either through M cells, via transepithelial sampling by mononuclear phagocytes, or by transcytosis of epithelial cells (5). Uptake of Ag, cell migration, and Ag presentation are critical steps in controlling systemic dissemination of the pathogen and induction of adaptive immunity (6). However, the cellular mechanisms used by this intestinal pathogen to move from the site of infection to the mesenteric lymph nodes (MLN) remain ill defined. In this study, we use two previously characterized oral infection protocols, with commonly used variants of STM, to understand this important immunological process.

Using the accurate definitions of murine intestinal mononuclear phagocytes that have now been developed (7, 8), we aimed to investigate which APCs are involved in the early events after oral STM infection and how the bacteria travel to the MLN, a critical tissue that limits systemic spread of STM (9). In this study, we find that although few cellular changes occur in the intestine in the days immediately following STM infection, bacteria can be detected migrating in cell-associated form and autonomously from the intestine to the MLN in intestine-draining lymph. The majority of the migrating bacteria are captured by macrophages in the MLN. Collectively, our results provide a detailed description of how *Salmonella* affects the intestine after the initial infection and uses the lymph to migrate to the MLN. Understanding this movement of STM to the MLN enables elucidation of the role of the various populations of APCs in controlling infection and initiating adaptive immune responses to intestinal bacteria.

## Materials and Methods

### Mice

C57BL/6 male mice were purchased from Envigo (Bicester, U.K.) and maintained in individually ventilated cages. CX3CR1^+/GFP^ reporter mice (originally a gift from Steffen Jung) were bred and maintained under specific pathogen-free conditions at the Central Research Facility, University of Glasgow, U.K.

### Ethics statement

All procedures were approved by the Animal Welfare Ethical Review Board at the University of Glasgow. This committee approved the project license, which was also approved by the U.K. Home Office. The project license has the number 60/4500. The license complies with the Animals (Scientific Procedures) Act 1986. The Animals (Scientific Procedures) Act has been revised to transpose European Directive 2010/63/EU on the protection of animals used for scientific purposes.

### Reagents

Cells were cultured in RPMI 1640, supplemented with 100 U ml^−1^ penicillin, 100 mg ml^−1^ streptomycin, 2 mM l-glutamine, 5% FCS (Invitrogen, Waltham, MA), and 50 mM 2-ME (Sigma-Aldrich, St. Louis, MO).

### Abs

Fluorochrome-conjugated mAb to mouse Ags CD11c on PE/Cy7, CD103 on APC, CD11b and MHC class II (MHC II) on AF700, MHC II and CD45 on V450, B220 on BV510, CD11b, streptavidin on BV605, and CD3 and CD64 on BV711 were from BioLegend (San Diego, CA).

### Surgical procedures

Animals were maintained under inhalation anesthesia with isoflurane (Abbott Labs, Abbott Park, IL). MLNx and thoracic duct cannulations were performed according to previously established protocols (10, 11). Briefly, mesenteric lymphadenectomy was performed on six-wk-old C57/BL6 male mice by laparotomy and blunt dissection. Six weeks later, mCherry STM-infected mice were fed 0.3 ml of olive oil to visualize the lymphatics, and the thoracic duct was cannulated by the insertion of a polyurethane cannula (2Fr; Linton Instrumentation, Diss, U.K.). Lymph was collected in PBS with 20 U ml^−1^ of heparin sodium (Wockhardt UK, Wrexham, U.K.) on ice for up to 16 h.

### Bacterial culture

All STM variants used were derived from the wild-type STM strain SL1344. Attenuated strain *ΔinvG* STM was kindly provided by Adam Cunningham (University of Birmingham), and *ΔaroA* pET-tac mCherry STM was kindly provided by Ian Henderson (University of Birmingham). For infection, a single bacterial colony was grown in Luria–Bertani (LB) broth at 37°C and 200 rpm before being back-diluted 1:10 in fresh LB broth and cultured in a static incubator overnight at 37°C.

### Infection model

Mice were pretreated with one of two of the following antibiotic regimens: a mixture of vancomycin (0.5 mg ml^−1^), neomycin (1 mg ml^−1^), metronidazole (1 mg ml^−1^), and ampicillin (1 mg ml^−1^) added to drinking water for 14 d prior to infection with Δ*invG* and Δ*aroA* mutants, or a single dose of streptomycin (20 mg) administered by oral gavage 24 h prior to infection with SL1344 (12). Mice infected by oral gavage with Δ*invG* STM received 1 × 10^8^ CFU, whereas pET-tac mCherry STM and SL1344 STM were adjusted to 5 × 10^7^ CFU.

### Cell isolation

Small intestine (SI) and colons were washed in HBSS with 2% FCS, following Peyer patch excision. Intestines were opened longitudinally and cut into 0.5 cm segments and incubated in HBSS with 2 mM EDTA (Sigma-Aldrich) at 37°C, shaking for 20 min to remove the epithelial cell layer and mucus. The SI lamina propria (LP) was digested with 1 mg ml^−1^ collagenase VIII (Sigma-Aldrich) for 20 min, whereas colonic LP was digested with 0.85 mg ml^−1^ of collagenase V (Sigma-Aldrich), 1.25 mg ml^−1^ collagenase D (Roche, Penzberg, Germany), 1 mg ml^−1^ dispase (Life Technologies, Paisley, U.K.), and 30 mg ml^−1^ DNase (Roche) for 40 min. Cells were then passed through a 40-μm cell strainer (VWR, Radnor, PA) and stained for flow cytometry. LNs were separated and incubated with 0.4 Wunsch units ml^−1^ Liberase TM and 50 mg ml^−1^ of DNase (Roche) for 45 min at 37°C while shaking. Single-cell suspensions were passed through a 40-μm cell strainer and stained for flow cytometry. Lymph from thoracic duct cannulation was filtered through a 100-μm mesh, washed with HBSS EDTA, and spun at 400 × *g* for 5 min. Next, the cellular pellet was separated from the supernatant, and this was spun once more, this time at 6500 × *g* for 5 min to pellet and collect cell-independent bacteria.

### Flow cytometry

Cell surface staining was performed in PBS with 2% FCS and 2 mM EDTA for 30 min on ice. To determine cell viability, 20 μl of 7AAD (BioLegend) was added to samples just before analysis, or 1 ml of fixable viability dye eFluor 780 (eBioscience) was added to samples in PBS for 30 min at 4°C before staining with Abs. Samples were acquired on an LSR II or a FACSAria III (BD Biosciences).

### Bacterial recovery

Spleen, SI, colon, MLN (for non-MLNx animals), and inguinal lymph nodes (ILNs) were harvested and weighed. Next, organs were homogenized using a stomacher, and bacteria present in tissues were quantified by plating of serial dilutions on LB agar. LB agar was supplemented with streptomycin 50 μg ml^−1^ for SL1344 and ampicillin 100 μg ml^−1^ for the experiments using mCherry STM.

### Statistical analysis

Data were analyzed using one- or two-way ANOVA and unpaired Student *t* test. The *p* values < 0.05 were considered significant. Statistical analysis was performed using Prism 6 (GraphPad, San Diego, CA).

## Results

### MLN dendritic cell numbers remain stable after antibiotic administration or STM infection

**FIGURE 1.**
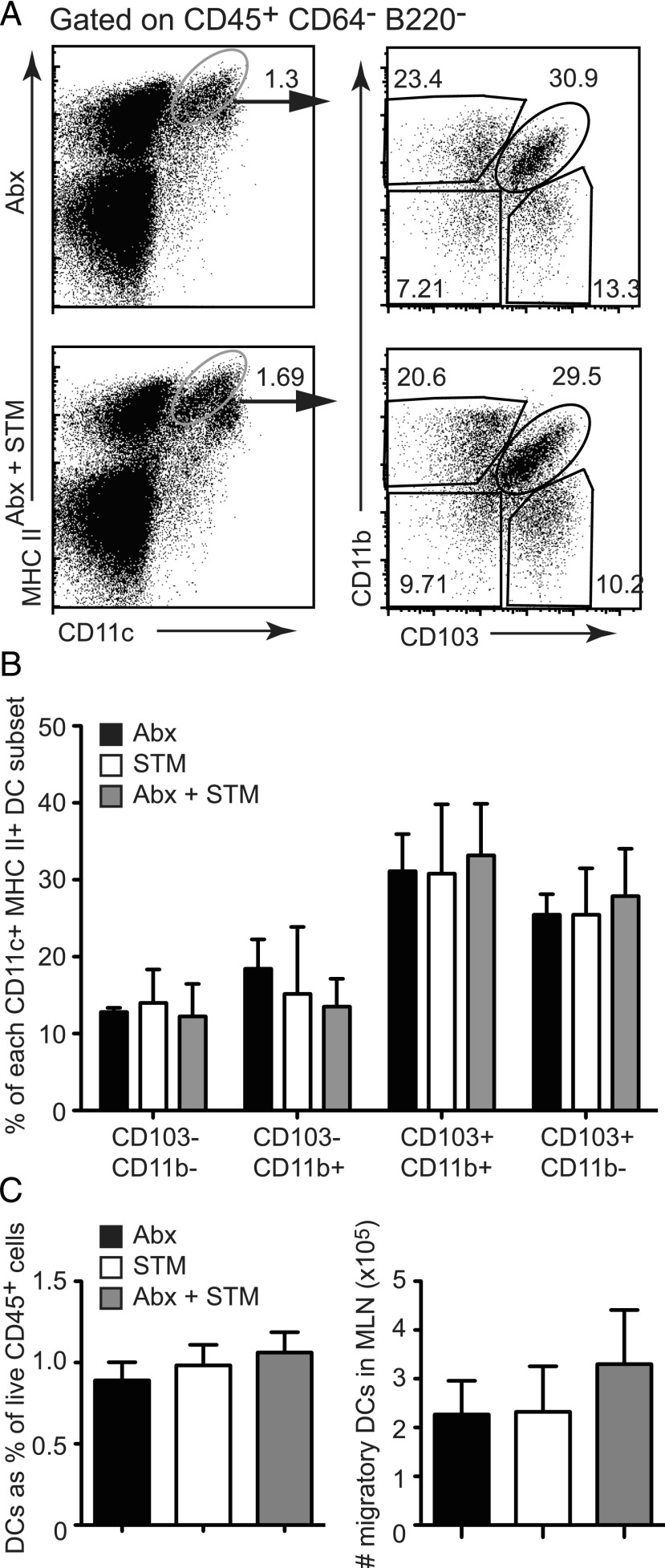
MLN DC populations are not altered by STM infection. (**A**) Migratory DCs (CD45^+^CD64^−^B220^−^MHC II^hi^CD11c^+^) from MLN of STM-infected or -uninfected mice (left panels) were categorized into four subsets based on CD11b and CD103 expression (CD103^+^CD11b^−^, CD103^−^CD11b^+^, double positive or double negative, right panels). Mice either received an antibiotic mixture alone (Abx) followed by 1 × 10^8^ ΔinvG STM, STM alone, or a combination of both. The frequency of each DC subset (**B**) and the numbers and frequencies of migratory DCs were calculated in each condition (**C**) (*n* = 4–6 per group, data representative of three independent experiments). Error bars represent SD.

We and others have previously shown that the dendritic cells (DCs) that migrate constitutively from the intestinal mucosa are known to transport Ag to the MLN (13–17). Migratory DCs are also able to transport *Salmonella* from the intestine, as demonstrated using *Salmonella* strains SL1344 and Δ*aroA*, an attenuated, auxotrophic SL1344 derivative (9). Interestingly, when an invasion-deficient attenuated STM strain (*ΔinvA*) was used, migrating macrophage-like cells were reported to be responsible for transporting STM from the intestine. Thus, to understand the effects of STM infection on migration, we first assessed the effect of STM infection on migratory cells in the MLN. To achieve bacterial colonization of the intestinal mucosa, animals were pretreated for 10 d with an antibiotic mixture, comprising vancomycin, neomycin, metronidazole, and ampicillin, or normal drinking water (negative control), which has previously been used to enable STM infection in experiments where macrophage-like cells were reported to migrate from the intestine (18, 19). Mice were orally infected with 1 × 10^8^ CFU of attenuated Δ*invG* STM. MLN were harvested 48 h postinfection (hpi), digested into single-cell suspensions, and analyzed by flow cytometry. The four populations of CD64^−^B220^−^CD11c^+^MHC II^hi^ migratory DCs, as previously described (20), were identified by their expression of CD103 and CD11b (CD103^+^CD11b^−^, CD103^−^CD11b^+^, CD103^+^CD11b^+^ double positive, or CD103^−^CD11b^−^ double negative) (Fig. 1A). No significant changes were observed in the proportions of these populations after antibiotic pretreatment, STM infection, or both (Fig. 1B). Similarly, no significant changes were observed in the frequency of migratory DCs and macrophage-like cells in the MLN or in the total number of migratory MLN DCs (Fig. 1C and data not shown). Thus, neither antibiotic pretreatment, nor STM infection, altered the frequency or number of any of the migratory DC populations in the MLN.

### STM infection induces increase in number of macrophages in the SI but not colonic LP

**FIGURE 2.**
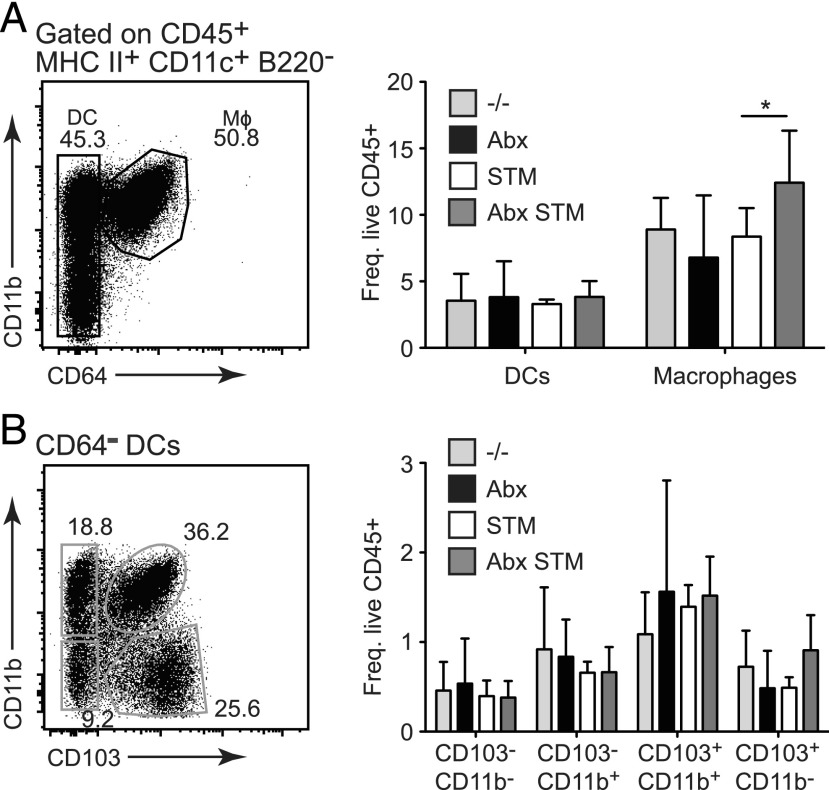
Antibiotics (Abx) and STM infection increase macrophage frequency in SI. Mice were pretreated with Abx mixture followed by oral infection with 1 × 10^8^ ΔinvG STM or gavaged with PBS. Forty-eight hpi, small intestines were harvested, and DC and macrophage populations were analyzed by flow cytometry. (**A**) Macrophages were identified from DCs by CD64 expression (left panel). SI LP was analyzed for total DC and macrophage frequencies and the frequencies of each of the DC subsets (**B**). *n* = 4–8 mice per group, representative of three independent experiments. Error bars represent SD. (A) Right panel, *p < 0.05, two-way ANOVA multiple comparisons.

At early time points postinfection, STM typically infects the SI by invasion of the M cells located in the Peyer patches (21). After antibiotic treatment, STM also colonize the cecum and colon (12). To understand the effects of STM infection on intestinal macrophage and DCs populations, we assessed their numbers in the SI and colon postinfection. We again used Δ*invG* STM so that we could compare our results with the previous (18). Mice were orally infected or given PBS after 2 wk pretreatment with the antibiotic mixture. This was followed by SI and colonic LP harvest at 48 hpi, followed by digestion into single-cell suspensions and analysis by flow cytometry. Mononuclear phagocytes were gated as live CD45^+^MHC II^+^CD11c^+^B220^−^ cells, following which macrophages were identified by their expression of CD64. Similar to our MLN data, the frequency of total LP DCs (Fig. 2A) and the proportion of each of the DC subsets in the SI LP remained unaltered following STM infection and/or pretreatment with the antibiotic mixture (Fig. 2B). There was a small increase in the frequency of CD64^+^ macrophages in the SI LP of mice 48 hpi with STM following antibiotic mixture pretreatment compared with those administered only antibiotics or STM (Fig. 2A). Interestingly, this increase was not observed in the colonic LP, where no significant changes in the frequencies of CX3CR1^int^ or CX3CR1^hi^ populations were observed (Supplemental Fig. 1). These data indicate that the numbers of DCs in the intestinal LP and the MLN are not altered following treatment with the antibiotic mixture and/or STM infection, whereas the frequency of CD64^+^ macrophages increased to a small but significant degree in the SI LP but not in the colonic LP after a combination of both antibiotic pretreatment and STM infection.

### CX3CR1^hi^ cells do not migrate in lymph after STM infection

**FIGURE 3.**
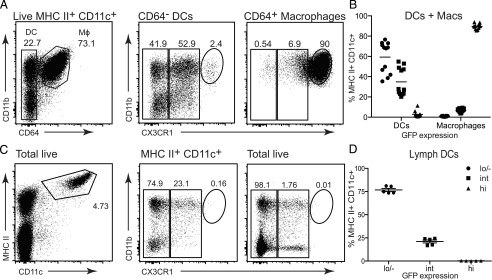
CX3CR1^hi^ cells do not migrate during STM infection. MLNx CX3CR1^+/GFP^ mice were infected with ΔinvG STM after pretreatment with the antibiotic mixture. (**A**) Representative FACS plots of CX3CR1 expression are shown, classified as low, intermediate, or high, on SI LP DCs (middle plot) and macrophages (right plot). The graph shows the percentage of DCs and macrophages with each level of CX3CR1 expression. (**B**) Frequency of intestinal DCs and macrophages being CX3CR1^lo/−^ (circles), CX3CR1^int^ (squares), and CX3CR1^hi^ (triangles). (**C**) CX3CR1 expression within total MHC II^+^CD11c^+^ lymph DCs (left and central plots) and total live lymph cells (right plot). (**D**) Frequency of lymph DCs being CX3CR1^lo/−^ (circles), CX3CR1^int^ (squares), and CX3CR1^hi^ (triangles). *n* = 4–6 per group, data pooled from two independent experiments.

Data from the MLN and intestine reveal only subtle changes in mononuclear phagocyte numbers induced by STM infection and antibiotic treatment. Therefore, to investigate whether STM infection affected cells migrating from the intestine to the MLN via lymphatics, we analyzed lymph cells directly obtained from the thoracic duct of lymphadenectomized (referred to as MLNx) mice. Normally, DCs migrating from the intestine complete their life cycle by performing their functions and ultimately dying in the MLN, hence the efferent lymph vessels are largely devoid of DCs. Thus, using previously developed techniques from our laboratory, MLN are surgically removed. Animals make a full recovery over a period of at least 6 wk, which allows reanastomosis of lymphatics and subsequent collection of “pseudo-afferent lymph” via cannulation of the thoracic duct (15). Following cannulation, animals recover from surgery and are returned to their cages; lymph is then collected for up to 24 h. Cannulation of CX3CR1^+/GFP^ MLNx mice not only allowed us to directly assess lymph-migrating cells but also to easily identify CX3CR1^hi^ mononuclear phagocytes, identified as macrophages (22), even at low numbers. MLNx CX3CR1^+/GFP^ mice were infected with the Δ*invG* STM after pretreatment with the antibiotic mixture used above (18). At 48 hpi, thoracic duct cannulation was performed and lymph collected. The SI was also harvested, processed, and analyzed for comparison. SI mononuclear phagocytes were identified as live MHC II^+^CD11c^+^ cells, and macrophages were differentiated from DCs by their CD64 expression (Fig. 3A, left plot). Intestinal DCs and macrophages in the CX3CR1^+/GFP^ mice expressed different levels of CX3CR1-GFP, which we describe as CX3CR1^lo^, CX3CR1^int^, or CX3CR1^hi^ (Fig. 3A, central and right panels, Fig. 3B). Our results show that, in our hands, only a residual fraction (<2.5%) of intestinal DCs were CX3CR1^hi^, whereas >90% of CD64^+^ macrophages expressed high levels of CX3CR1-GFP (Fig. 3A, 3B). Moreover, of the lymph cells harvested from the thoracic duct of MLNx mice postinfection, none were CD64^+^, and fewer than 0.2% fell within the CX3CR1^hi^ gate (Fig. 3C middle and data not shown). Almost a quarter (23%) of lymph DCs were CX3CR1^int^ (Fig. 3C, 3D). To confirm that no CX3CR1^hi^ cells from lymph had been excluded as a result of the flow cytometric gating on CD11c^+^MHC II^hi^ cells, total live cells in the lymph were also analyzed. These results mirrored the results from pregated lymph (Fig. 3C, right panel). Thus, although >90% of macrophages in the SI LP express high levels of CX3CR1, these cells are absent from lymph after STM infection. Contrary to previously published data using a similar strain of STM and similar antibiotic pretreatment (18), after STM infection, the CD11c^+^ MHC II^hi^ lymph migratory cells are DCs (CX3CR1^int^ or CX3CR1^lo^) rather than CD64^+^CX3CR1^hi^ macrophages.

### All DC subsets can carry fluorescent STM in lymph

**FIGURE 4.**
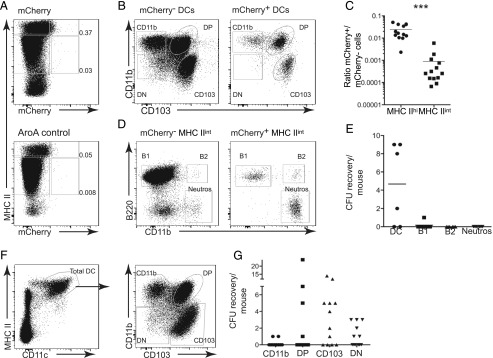
All DC subsets can carry viable STM. MLNx mice were infected with 1 × 10^8^ mCherry AroA STM or AroA STM control after pretreatment with an antibiotic mixture, and lymph was collected 48 hpi. (**A**) mCherry^+^ events were analyzed from among live lymph cells and subdivided based on their MHC II expression. (**B**) DC subsets within mCherry^−^ MHC II^hi^– and mCherry^+^ MHC II^hi^–infected lymph. (**C**) Relative abundance comparison between mCherry^+^ events in MHC II^hi^ and MHC II^int^ lymph fractions. (**D**) B220 and CD11b expression from mCherry^−^ and mCherry^+^ within MHC II^int^ cells. (**E**) AroA STM recovery from sorted total DC, B1, B2, and neutrophil populations. (**F**) AroA STM recovery from sorted DC subsets. (**G**) AroA STM viable colonies recovered per mouse from each lymph DC subset. Data representative of at least three independent experiments. ***p < 0.001, Wilcoxon matched pairs signed-rank test.

Having established that macrophage-like cells do not migrate in lymph after STM infection, we aimed to understand how STM migrate from the intestine to the MLN. To achieve this aim, we switched to the commonly used auxotrophic SL1344-derived Δ*aroA* mutant (23). Use of this auxotrophic strain enables analysis of STM uptake and transport independent of confounding factors that occur if extensive bacterial replication can occur. To positively identify the cells carrying STM in lymph in the initial period postinfection, we used an mCherry-expressing strain of Δ*aroA* STM. This allowed us to analyze cells associated with fluorescent bacteria by flow cytometry. After a 2-wk antibiotic pretreatment, MLNx mice were infected with mCherry STM, and lymph was collected 48 hpi for analysis. We expected that only MHC II^hi^ DCs would be associated with an mCherry-positive signal. However, our results revealed mCherry^+^ cells with at least two different levels of MHC II expression. These data indicate that multiple lymph-migrating cell types could be associated with STM (Fig. 4A). Because it is known that DCs express high levels of MHC II, including when they travel in the lymph (8, 24), we first analyzed mCherry^+^ and mCherry^−^ MHC II^hi^ cells to identify any dominant STM-transporting DC population. Our results showed that all DC populations were present in similar proportions among both the mCherry^+^ and mCherry^−^ cells (Fig. 4B). We calculated the ratio of mCherry^+^ over mCherry^−^ events and compared them between MHC II^hi^ and MHC II^int^ cells, thus demonstrating that DCs were the cells most frequently associated with mCherry STM (Fig. 4C). We also identified an mCherry^+^ signal within the MHC II^int^ population, which we investigated to characterize these populations (Fig. 4D). We observed that the mCherry^+^ MHC II^int^ population is composed of two B cell populations (B220^+^CD11b^−^ and B220^+^CD11b^+^) plus a third population mostly made up by B220^−^CD11b^+^Ly6G^+^ neutrophils. (Fig. 4D, Supplemental Fig. 2).

To determine whether the lymph cells carried live bacteria, we attempted to recover STM CFU from flow-sorted lymph cells grown on selective culture medium. Thus, we infected MLNx mice with conventional Δ*AroA STM* sorted up to 2.5 × 10^5^ total DCs and the three MHC II^int^ subsets. Sorted cells were then plated on streptomycin-enriched LB agar to select STM colonies (Fig. 4E). Interestingly, we only recovered viable Δ*AroA* STM from DCs. To then identify whether any particular DC subset carried STM, we flow-sorted and cultured each of the DC subsets (Fig. 4F). We find that CD103^+^CD11b^+^ and CD103^+^CD11b^−^ cells carried most of the STM, but some colonies were cultured from all DC subsets (Fig. 4G).

Taken together, our data indicate that although STM may be associated with B cells and neutrophils in lymph, only DCs carry viable STM.

### A proportion of STM migrates by a cell-independent mechanism

**FIGURE 5.**
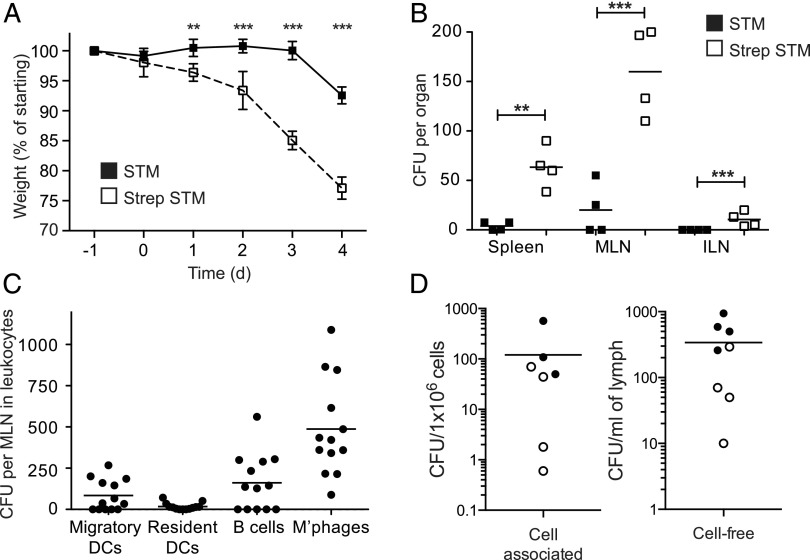
STM dissemination to the MLN is only partially dependent on cell-mediated transport. C57BL/6 mice were pretreated with either 20 mg of streptomycin (Strep) or PBS (control) followed by oral infection with 1.5 × 10^8^ CFU of SL1344 STM after 24 h [(**A** and **B**) *n* = 4 mice per group, data representative of two independent experiments]. Animals were weighed daily, and after 4 d, bacteria were recovered and quantified from spleen, MLN, and ILN. (**C**) APC populations from MLN of antibiotic-treated infected mice (5 × 10^7^ CFU) were sorted and plated in streptomycin-supplemented LB agar 48 hpi. CFUs were counted from migratory and MLN-resident DCs, B cells, and macrophages, and the total CFU per MLN were calculated (*n* = 3–5 mice per group, data pooled from three independent experiments). (**D**) Cell-associated and cell-free STM was harvested from lymph and quantified either from animals pretreated with an antibiotic mixture and administered 1 × 10^8^ CFU of ΔinvG STM (filled circles) or from animals pretreated with 20 mg of streptomycin and administered 5 × 10^7^ CFU of SL1344 STM (open circles) (*n* = 2–4 mice per group, data representative of at least three experiments). Error bars show SD. (A) **p < 0.01, ***p < 0.001, two-way ANOVA with multiple comparisons. (B) **p < 0.01, ***p < 0.001, one-way ANOVA.

Results above were obtained using attenuated auxotrophic STM strains. To assess whether nonattenuated STM is also transported by similar mechanisms, we performed the following experiments with the SL1344 parent strain (12, 24, 25). As is common for experiments with SL1344, mice were pretreated with a single oral dose of 20 mg of streptomycin 24 h before oral infection to increase the numbers of colonizing STM. By 96 hpi, streptomycin-treated STM-infected animals had, as expected, experienced significant weight loss compared with STM-infected animals (Fig. 5A). To assess the extent of dissemination of STM in these animals, we harvested spleens, MLN, and ILN and quantified STM CFUs by homogenizing and plating tissue on streptomycin-enriched LB agar. As expected, infected animals that were treated with streptomycin yielded significantly higher CFUs in the MLN than animals not pretreated with streptomycin. Similar to the antibiotic mixture used above, streptomycin pretreatment did not affect the frequency or numbers of macrophages and DCs in the intestinal LP or MLN (Supplemental Fig. 3). Systemic spread of STM was observed in streptomycin-pretreated mice, indicated by their significantly higher CFU counts from spleen and ILNs (Fig. 5B). The final stages of the dissemination to the spleen must occur via the bloodstream, as the spleen lacks a lymphatic supply (26, 27). To assess the as-yet-unknown contribution of intestinal lymph to the transport of STM in this model, we first aimed to identify the cells in the MLN that become STM infected. From our data above, we predicted that migratory DCs in the MLN, which express the highest levels of MHC II, would harbor the highest numbers of STM. We therefore flow-sorted intestine-derived migratory DCs (MHC II^hi^CD11c^+^), MLN-resident DCs (MHC II^+^CD11c^+^), B cells (B220^+^), and macrophages (CD64^+^) from MLN of infected mice. Each of these purified populations was lysed with a detergent solution and plated overnight. As expected, STM was recovered from migratory DCs, whereas MLN-resident DCs yielded fewer CFUs (Fig. 5C). Interestingly, CD64^+^ macrophages in the MLN carried the highest number of CFUs despite the fact that they do not migrate in lymph (Fig. 3). Therefore, to understand how lymph node–resident macrophages acquire STM, especially when so few DCs are actively carrying live bacteria, we investigated whether STM may be traveling in lymph in a cell-free form and being captured by macrophages upon reaching the MLN. We tested this after pretreating animals with either the antibiotic mixture or streptomycin treatment. MLNx mice were pretreated with antibiotics and infected with SL1344 STM. Lymph was obtained by thoracic duct cannulation of MLNx mice, as described above, and cells were separated from the lymph supernatant by centrifugation. The cell pellet and lymph supernatant were plated separately and CFUs quantified. This revealed that, surprisingly, similar numbers of STM were detected in the cell-associated and cell-free lymph fractions (Fig. 5D). Taken together, our results demonstrate that no macrophages travel in lymph, small numbers of viable bacteria are carried by migratory lymph DCs, and STM also travel in lymph independently of cells and are delivered to macrophages and B cells in the MLN.

## Discussion

The early immune response to STM infection and the role of the MLN in limiting bacterial dissemination have been well characterized (5, 9, 28). However, the involvement of the intestinal lymphatic drainage in bacterial migration from the intestinal mucosa to the MLN, to our knowledge, has not previously been clearly described. In this study, we used attenuated and nonattenuated STM to examine their effects on previously reported STM-associated DC and macrophage populations. We demonstrate that STM infection causes only subtle cellular disruptions in the days immediately postinfection. We then examined associations with cell populations from the MLN and thoracic duct lymph to understand how STM migrates to the MLN. Our work reveals that although STM may be associated with MHC II^int^ cells (B cells and neutrophils), viable bacteria are only transported by DCs and, surprisingly, by an autonomous route.

We initially demonstrated that the frequencies of DC populations do not change, either in the intestinal LP or the MLN in the 2 d after STM infection. Thus, the infection does not have significant early effects on the overall patterns of DC migration. We did observe, however, an increase in the frequency of macrophages in the SI of antibiotic-treated infected mice (Figs. 1, 2). This may reflect an influx of proinflammatory cells to the infected intestine. Although the colon and cecum are the prominent sites of pathology during infection, the SI may be the initial site of bacterial colonization (5). This colonization likely leads to increased monocyte infiltration, which then accumulate and develop into CD64^+^ macrophages (29).

A pivotal feature that differentiates DCs from macrophages is their ability to migrate to draining lymph nodes (14, 15). However, it has been reported that CX3CR1^hi^ mononuclear phagocytes are induced to migrate in lymph after STM infection, carrying bacteria to the MLN of infected mice (18). To characterize these migrating cells, we performed thoracic duct cannulation on STM-infected CX3CR1^+/GFP^ reporter mice, using a similar preinfection antibiotic treatment and STM strain as in the previous report. We demonstrate in this paper that DCs, which can express intermediate levels of CX3CR1 (15), were able to migrate in intestinal lymph, whereas none of the lymph-migrating cells expressed high levels of CX3CR1 or CD64. Thus, DCs, but not macrophages, travel from the intestine to the MLN in the first 48 h after STM infection.

To find which cells carry STM from the intestine to the MLN, we used Δ*aroA* mCherry-expressing STM, which can be detected by flow cytometry. We therefore collected the cells migrating in lymph after STM-mCherry infection of antibiotic-treated mice and quantified the frequencies of cells associated with mCherry^+^ fluorescent STM. We found the mCherry^+^ signal in lymph associated with all DC subsets. Interestingly, mCherry^+^ cells were also found among MHC II^int^ lymph cells, including two populations of B cells (B220^+^CD11b^−^ and B220^+^CD11b^+^) and neutrophils. Although we have previously observed small numbers of neutrophils in samples of lymph from cannulated animals, it had not been possible to determine whether these cells were from the lymph itself or from a tiny volume of contaminating blood. In this study, however, a considerable proportion of the neutrophils are mCherry^+^ after mice have been orally infected and before systemic STM are detectable; therefore, we think they are likely to originate from the intestine and travel to the MLN (26, 27).

To quantify live STM carried by each of the different mCherry^+^ cell types, we sorted and plated cells on selective medium. This revealed that viable STM is carried exclusively by DCs. Although slightly more STM was recovered from CD103^+^CD11b^+^ and CD103^+^CD11b^−^ DCs, all DC populations have the ability to carry viable bacteria. Calculations from these STM culture experiments reveal that only approximately one in 47,000 DCs carries viable bacteria. Thus, we sought to understand whether there may be other routes by which viable bacteria reach the MLN.

Strikingly, as well as observing that STM travels in lymph in DCs, we found that only half of the STM in lymph are traveling in cell-associated form. This autonomous migration of STM in intestinal lymph could explain why we recover more STM CFUs from MLN-resident macrophages and B cells than from MLN migratory DCs. The STM-positive macrophages are likely to be macrophages lining the subcapsular sinuses that are known to bind extracellular pathogens from lymph (30, 31). As the macrophages themselves do not migrate in lymph, the STM-associated macrophages in the MLN may have acquired many of the bacteria that arrive in the MLN via lymph in a cell-free form (32). Finally, a recent report showed that mesenchymal cell MyD88-dependent sensing of STM may contribute to its systemic spread (33), thus highlighting the importance of the MLN and its resident cells and raising new questions about the potential functions of MLN.

Our data demonstrate that the migration of STM from the intestine to the MLN via lymph is more complex than previously thought. We reveal that in lymph, viable STM is not only transported by DCs but also autonomously. On reaching the MLN, STM is taken up by MLN-resident macrophages, DCs, and by some B cells.

## Supplementary Material

Data Supplement
