# Supplementary material for: Salmonella enterica Serovar Typhimurium Travels to Mesenteric Lymph Nodes Both with Host Cells and Autonomously
Source: J Immunol. 2018 Nov 28;202(1):260–7. doi: 10.4049/jimmunol.1701254 (PMC6305795; doi:10.4049/jimmunol.1701254)
Supplement: Data Supplement [file JI_1701254.zip › JI_1701254_Supplemental_Figures_1.pdf]

Supplementary figure 1

A

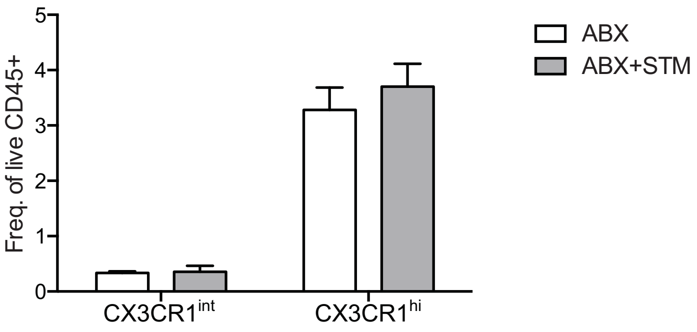

Supplementary figure 1. Mice were pre-treated with Abx cocktail followed by oral infection with $1x10^8$ ΔinvG STM or gavaged PBS. 48 hpi, frequency of colonic LP CX3CR1-expressing cells were calculated. Error bars represent standard deviations. n=4 mice per group.

Supplementary figure 2

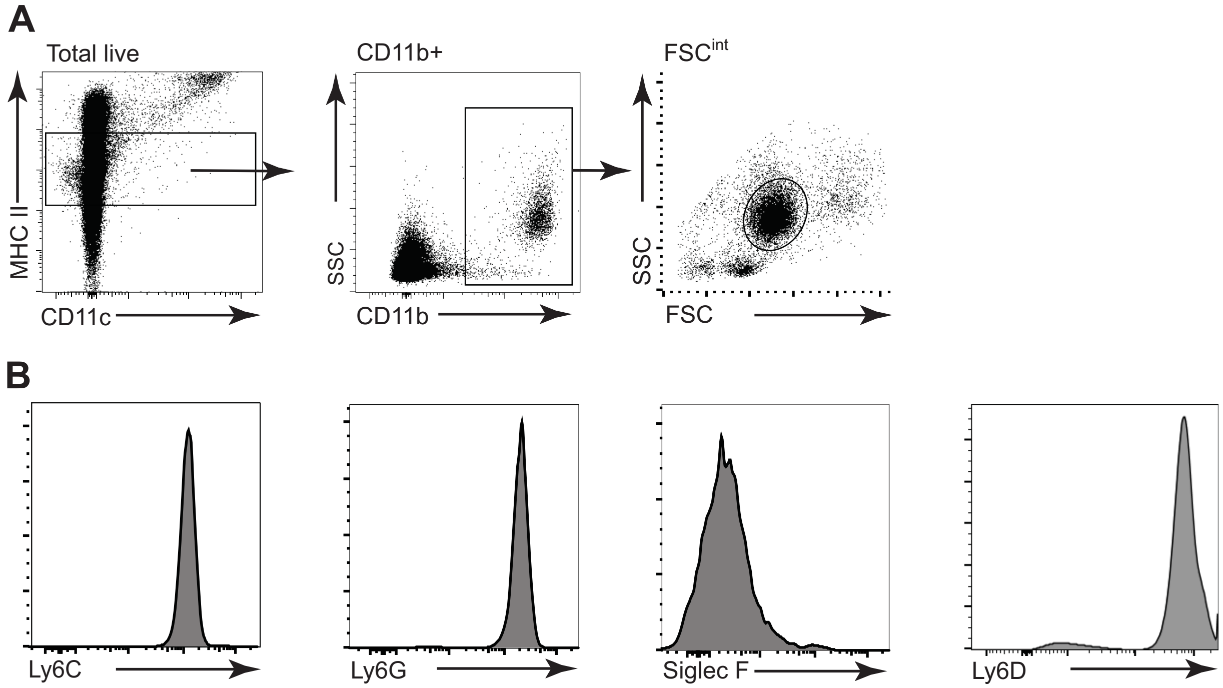

Supplementary figure 2. Lymph was harvested via thoracic duct cannulation from MLNx mice. (A) Representative FACS plots of MHC II^{int} cells and their gating based on CD11b, SSC and FSC properties and (B) showing expression of Ly6C, Ly6G, siglec F and Ly6D. Data representative of at least 3 independent experiments.

Supplementary figure 3

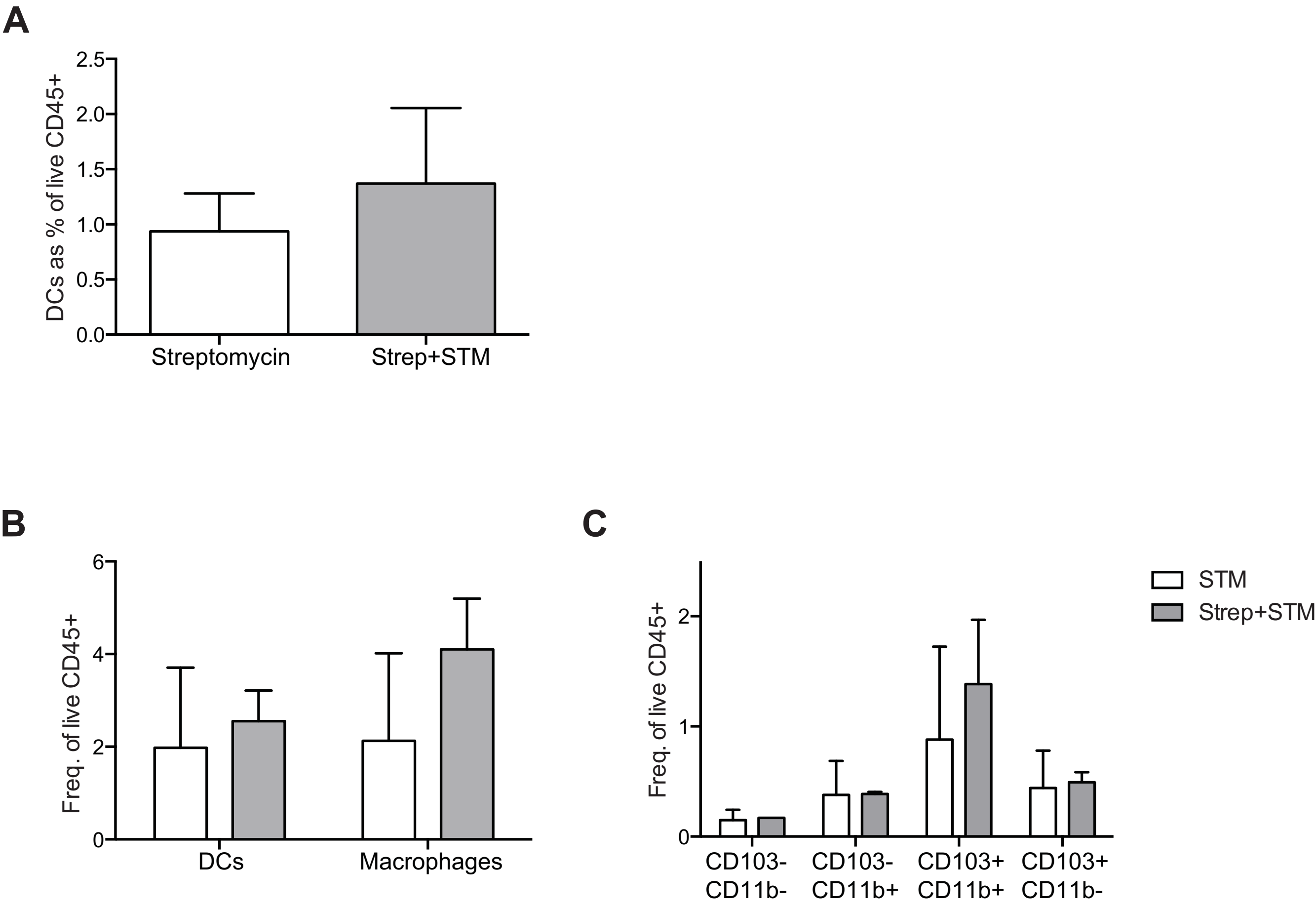

Supplementary figure 3. (A) Streptomycin pre-treated mice (20 mg) were orally infected 24 hpi with 5x10^7 CFU SL1344 STM (Strep+STM) or gavaged with PBS and frequency of total DCs from MLN were measured (n=3-4 mice per group). (B) Frequency of small intestine LP total DCs and macrophages from mice infected with or without streptomycin pre-treatment (n=4 mice per group). (C) Frequency of small intestine LP DC subsets from mice infected with or without streptomycin pre-treatment (4 mice per group).
